# Dr. West on Modesty

**Published:** 1892-07

**Authors:** 


### DR. WEST ON MODESTY.

### HIS REPLY TO "RETRENCH OR BANKRUPT."

The editorial in our June number which has thrown Secretary West into such paroxysms of wrath and virtuous indignation, was by no means intended as a reflection either on his integrity or his intentions. It was sought to show that however "willing to pay a little more (?) for a 'decent' volume" the Association may be, by reason of the slim attendance and accessions at Tyler, and the consequent slim receipts, it is in no condition to afford another volume of Transactions at such a cost as that of last year. It was also sought to illustrate by actual figures the fact that the same work could have been done elsewhere than in Galveston, for several hundred dollars less money, had Dr. West allowed other printers to bid on the contract. According to the doctor's admission in his "open letter" published herewith, the volume of

1891–2 cost 35 cents per copy more than that of the previous year, notwithstanding the latter contained sixty-six pages more, and, we hold, was equally as good. Under the management of the old publishing committee at Austin, the Transactions were gotten out six consecutive years; four years, uniform, in purple cloth and gilt. The volumes of 1888 and 1889, when the finances of the Association were cramped, were issued in paper. These volumes all gave very general satisfaction. Why, then, pay forty odd per cent. more to have it printed in Galveston, and refuse all offers of competition? The old committee held that it was due the Association that they should have the best work for the least money; hence, each year, they advertised for bids, and often received them from Houston, San Antonio, Dallas, Fort Worth, and even from New Orleans and Chicago. Von Boeckmann, at Austin, secured the contract for several consecutive years—giving bond and security for the performance of the agreement.

Dr. West thinks Dr. Daniel's offer to assist him in proof-reading, etc., should the contract fall to Austin, was immodest. Some people have strange ways of looking at things and queer ideas of modesty and courtesy. We still think that some three or four hundred dollars could have been saved had the committee advertised for, or, even accepted, bids from other printers; and this year—from indications at Tyler—it will not be a matter of choice, but of necessity, that the Transactions shall be less expensive.

The good doctor is surely jesting when he says that the Transactions issued at Austin were the worst of any State from Maine to California. The following press opinions of the Texas Transactions were reproduced in the report of the Publishing Committee in 1887, and will be found on page 38 of the Transactions for that year. The volumes of 1885, '86, '87, and '90 were uniform:

> It far exceeds in general make up the average Transactions from any of our State Societies, . . . well written and well edited.—*Miss. Valley Med. Monthly.*

> The paper, press work and binding are models of good taste. —*Amer. Lancet.*

> . . . in every way the most creditable work of its kind in this country. No other State can offer anything for favorable comparison with it.—*Progress, Louisville, Ky.*

> . . . handsomely bound and printed. It is one of the most complete volumes of the kind we have ever seen; and in the appearance of its Transactions the Texas State Medical Association holds an easy lead—*Independent Practitioner*, *Buffalo, N. Y.*

The Texas State Medical Association and its committee of publication are to be congratulated on one of the handsomest and most elegant volumes of Society Transactions that we have ever seen . . . . it is rather a stunner, and will compare more than favorably with those from more intellectual (?) localities on this continent, or eveu in "Yurrup." The handsome cloth binding, elegant paper, and neat typography would be a credit to the most enterprising and completely equipped publishing house of either continent.—*Southern Practitioner*, *Louisville.*

The volume of Transactions issued by the Texas Association last year has been hailed everywhere as a work of merit.—*N. O. Med. and Surg. Journal.*

Dr. West stultifies himself when he essays to belittle the work of his predecessors, in the face of such commendation.

The stenographer was employed under a resolution of Dr. West, the last month of Dr. Daniel's term, especially to report the discussions. Dr. D. never had the benefit of his services or those of any other stenographer. In this Dr. West is unjust in his strictures. The stenographer did more for Dr. West—he took down and transcribed the minutes,—work always done by his predecessor, and for which, in part at least, the salary of $200 is paid the Secretary.

Dr. West has failed to show wherein our figures have "lied," even constructively. They were taken from the record and were correct. He calls attention to the fact that "$500 of that vast sum went into Dr. Daniel's pocket." Very true; so it did, each year, —*at the end of the year*, and when his work *had all been done*, was submitted *and approved*; it was one of those "usual items of disbursements" which, we said, appeared each year in the Treasurer's report.

But we overlooked the fact that there was *one very unusual* "item of disbursement" in the Treasurer's report—the new Secretary (West's) salary—*in advance.* Dr. West was elected on the 28th of April, '92. On the 28th of July, '92,—just ninety days after being inducted into office, and "before he had gotten warm in his seat," drew his entire salary—$200 as Secretary, and $300 as chairman of publishing committee—in full,—for work *to be done!* And yet (we cannot suppress the reflection—it comes like an echo of Waco), the Doctor said: "I didn't want the office. . . . I have been selected as the exponent of peace and progress. I am the man upon whom you all look in the office of Secretary to promote the welfare and good feeling in the

Association . . . It is because I love the Association . . that I accept the office." . .

Of course, it is easily to be seen that the salary was no object; it was drawn in advance only to get it off of his mind. "Verily thy name is modesty" oh West! Thou art the only Secretary the Association ever had who possessed the unparalleled—modesty—to ask for pay before it had been earned. This is a bad precedent. In case of death somebody else would have had to be paid another five hundred to do the work. Figure-head? Not a bit of it!

Well, it is no funeral of ours, and having sounded the note of warning, and indulged in these few remarks in self defense we "stand from under." We have spoken.

